# Id proteins: emerging roles in CNS disease and targets for modifying neural stemcell behavior

**DOI:** 10.1007/s00441-021-03490-z

**Published:** 2021-07-24

**Authors:** Yu-Hsuan Chu, Jia-di Lin, Suvra Nath, Christian Schachtrup

**Affiliations:** 1 Institute of Anatomy and Cell Biology, Faculty of Medicine, University of Freiburg, Freiburg, Germany; 2 Faculty of Biology, University of Freiburg, Freiburg, Germany; 3 Center for Basics in NeuroModulation (NeuroModulBasics), Faculty of Medicine, University of Freiburg, Freiburg, Germany

**Keywords:** Helix-loop-helix transcription factor, Extracellular matrix, Fibrinogen, Multiple sclerosis, Small molecule inhibitors

## Abstract

Neural stem/progenitor cells (NSPCs) are found in the adult brain and spinal cord, and endogenous or transplanted NSPCs contribute to repair processes and regulate immune responses in the CNS. However, the molecular mechanisms of NSPC survival and integration as well as their fate determination and functionality are still poorly understood. Inhibitor of DNA binding (Id) proteins are increasingly recognized as key determinants of NSPC fate specification. Id proteins act by antagonizing the DNA-binding activity of basic helix-loop-helix (bHLH) transcription factors, and the balance of Id and bHLH proteins determines cell fate decisions in numerous cell types and developmental stages. Id proteins are central in responses to environmental changes, as they occur in CNS injury and disease, and cellular responses in adult NSPCs implicate Id proteins as prime candidates for manipulating stemcell behavior. Here, we outline recent advances in understanding Id protein pleiotropic functions in CNS diseases and propose an integrated view of Id proteins and their promise as potential targets in modifying stemcell behavior to ameliorate CNS disease.

## Introduction

Neurological diseases and injuries to the adult mammalian central nervous system (CNS) often lead to permanent cell loss and functional impairment because the CNS has only a limited regeneration capacity. Therapeutic strategies based on neural stem/progenitor cells (NSPCs) are of great interest for promoting CNS regeneration. These approaches work by activating the tissue-resident stemcell reservoir or providing exogenous NSPCs as an alternative source (Assinck et al. 2017; Fischer et al. 2020; Llorens-Bobadilla et al. 2020; Lu et al. 2012; Peruzzotti-Jametti et al. 2018; Pluchino et al. 2020). The contributions of NSPCs to cell replacement and their ability to mediate immune responses are well recognized. However, the molecular mechanisms that regulate NSPC survival, cell-fate determination, and functional integration into the injured tissue under pathological conditions are poorly understood.

A comprehensive understanding of these regulatory paths would be valuable for harnessing both endogenous and transplanted NSPCs for a more effective therapeutic repair. A fine-tuned cellular and molecular environment of NSPCs is instrumental for maintaining and directing their differentiation (Chaker et al. 2016; Ihrie and Alvarez-Buylla 2011; Schildge et al. 2014). Pathological states alter the finely tuned NSPC environment by disrupting signaling pathways and transcriptional networks resulting in misguided NSPC fate and functionality and thus limiting the NSPC potential for CNS regeneration.

**Fig. 1:**
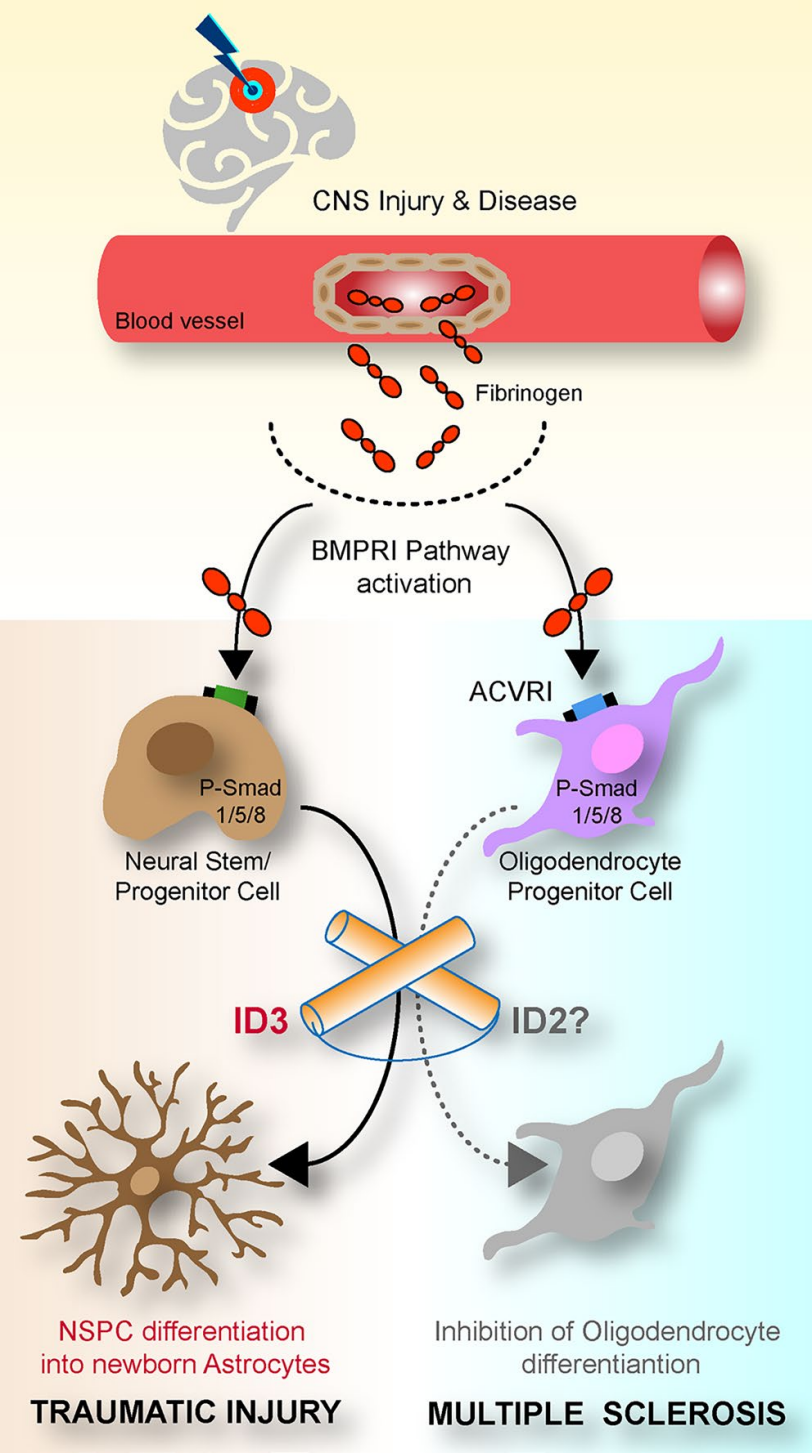
Id proteins regulate NSPC and oligodendrocyte progenitor-cell fate in CNS injury and disease. After traumatic injury (left) or multiple sclerosis (right) with BBB opening or vascular rupture, blood-derived fibrinogen activates BMP signaling. As a result, Id upregulation in NSPCs and oligodendrocyte progenitor cells induce astrogliogenesis and inhibit oligodendrocyte differentiation, respectively

Recently, our laboratory identified inhibitor of DNA (Id) proteins as a link between the altered cell environment and the ability of transcription factors (TFs) in NSPCs to regulate NSPC differentiation and functionality. We showed that blood-derived fibrinogen is enriched in the NSPC environment after CNS injury (e.g., traumatic brain injury) or neurological disease (e.g., multiple sclerosis (MS)). Mechanistically, fibrinogen activates bone morphogenetic protein (BMP) type I receptor (BMPR I) signaling and triggers Id expression in NSPCs and oligodendrocyte progenitor cells (OPCs), inducing astrogliogenesis and inhibiting oligodendrocyte differentiation, respectively (Lin et al. 2021; Petersen et al. 2017; Pous et al. 2020) (Fig. 1). Thus, we found that, as the stemcell microenvironment changed during disease states, the BMPR I–Id axis responds rapidly to orchestrate NSPC behavior. Here, we provide an overview on the biological properties of Id proteins and discuss the current knowledge about the Id function in CNS disease and how this knowledge might be applied to modify NSPC fate and functions that promote CNS repair.

## Id structure and interaction partner

**Fig. 2:**
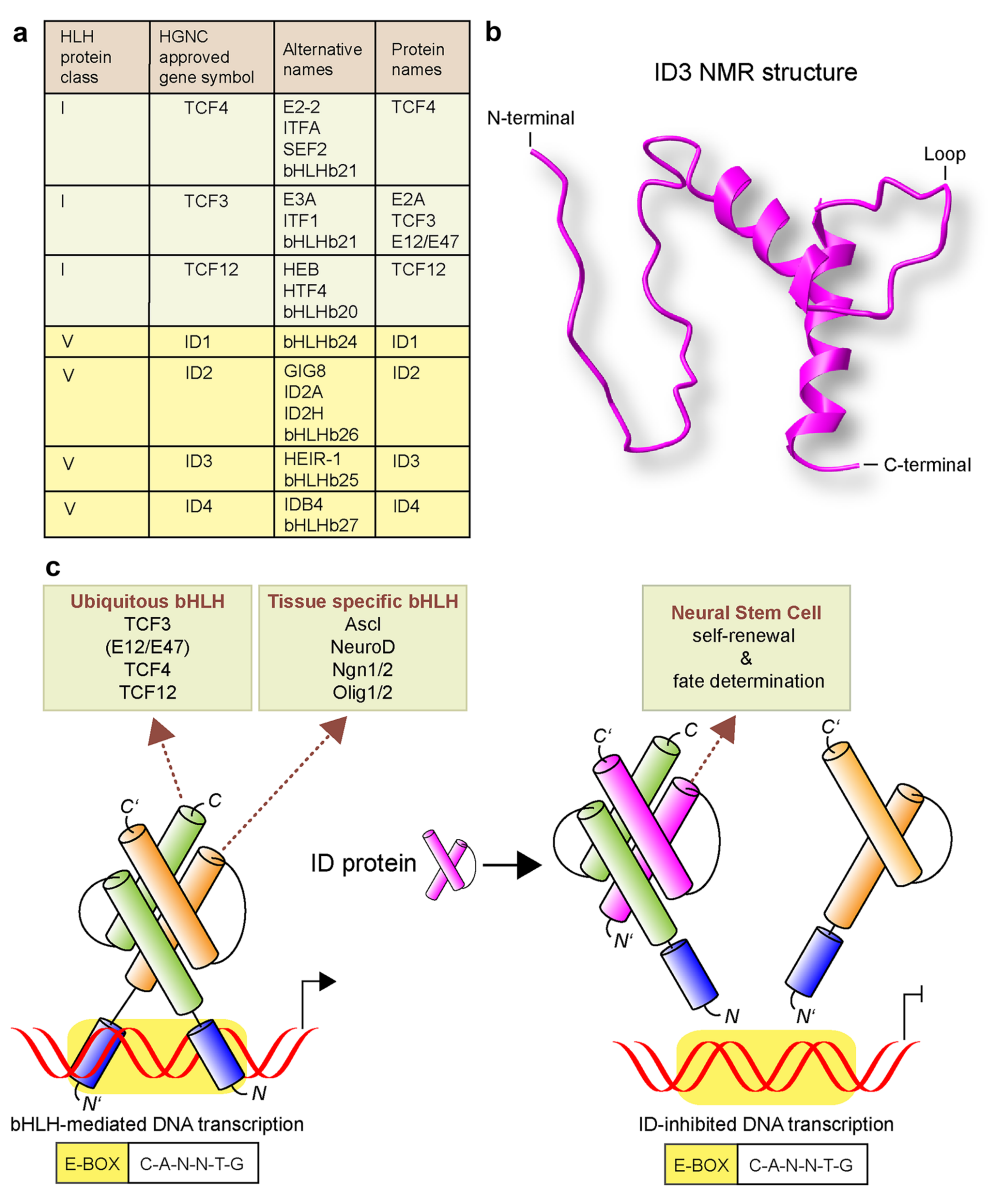
Id protein control of NSPC self-renewal and differentiation. **a** Class I and class V proteins of the HLH transcription factor family. **b** NMR structure of Id3 (UniProtKB). **c** Ubiquitously expressed bHLH E proteins build heterodimers with tissue-specific bHLH proteins, which results in DNA binding at specific DNA sequences (E-box in yellow) and DNA-transcription activation. Dimers of E proteins and Id proteins cannot bind DNA because the Id subunit lacks a DNA binding region, which leads to inhibition of DNA-transcription activation

Id proteins (Id1–Id4) belong to the helix-loop-helix (HLH) protein family (Fig. 2a) (Murre 2019). Each of them contains a highly conserved HLH domain, but lacks a basic region for DNA binding (Fig. 2b). Thus, the crucial biochemical attribute of each Id family member is their antagonizing effect on basic (b) HLH TFs that orchestrate cellfate determination (Dennis et al. 2019). The bHLH domain is tripartite and comprises a basic DNA-binding region and two amphipathic α-helices connected by a loop domain. Binding occurs through the basic region adjacent to the HLH motif, and they bind to target genes with a distinct DNA segment, named E-box site, that contains a signature core of six nucleotides: CANNTG. Lineage-restricted class II bHLH TFs, including the neural-specific bHLH TFs Ascl, NeuroD, Ngn1/2, and Olig1/2, form heterodimers through the HLH dimerization region with ubiquitously expressed class I (E protein) bHLH proteins (e.g., E2A/TCF3, E2-2/TCF4, and HEB/TCF12) (Bertrand et al. 2002). The primary binding partners of Id proteins are class I bHLH proteins (E proteins) (Langlands et al. 1997). In the presence of excessive Id proteins, Id-bHLH rather than bHLH–bHLH association prevails, and because the Id proteins do not contain a basic region, the Id-bHLH heterodimer is unable to bind to DNA, and bHLH-directed transcription is blocked (Fig. 2c) (Norton et al. 1998; Sun et al. 1991).

The four Id proteins are characterized by an HLH motif of 41 residues and by different long N- and C-terminal tails. Although the HLH domain of the Id proteins is involved in most of their protein–protein interaction events, additional motifs located in their N-terminal and C-terminal regions are required for the recognition of diverse protein partners. The ability of the Id proteins to interact with structurally different proteins is likely to arise from their conformational flexibility. Indeed, these proteins contain intrinsically disordered regions (Beisswenger and Cabrele 2014; Colombo and Cabrele 2006; Kiewitz and Cabrele 2005; Linding et al. 2003; Obradovic et al. 2005) that, in the case of the HLH region, undergo folding upon self- or heteroassociation, while the N-terminal and C-terminal regions maintain a high degree of flexibility (Beisswenger et al. 2010; Eletsky et al. 2011; Kiewitz et al. 2008; Svobodova and Cabrele 2006; Wong et al. 2012). While the identity between the HLH regions is very high for the four Id proteins, their other parts are not conserved (Kiewitz and Cabrele 2005; Roschger and Cabrele 2017), and these sequence differences explain the differences in protein structure and stability, subcellular localization, binding partner, and subsequent functionality between Ids. For example, a destruction box motif (D-box) is conserved in Id1, Id2, and Id4, but not in Id3 (Lasorella, et al. 2006); but a nuclear export signal (NES) is only found in Id2 (Karaya, et al. 2005) and Id1 (Makita et al. 2006), suggesting differential protein degradation and regulation of the subcellular distribution of Ids. For example, Id2 is the only Id protein to recognize the retinoblastoma protein, presumably by interactions between the HLH region and the B-box of the cyclin fold (Iavarone et al. 1994; Lasorella et al. 2000). Furthermore, the potency order of Id2 binding to the ubiquitous TFs is as follows: E47 > E12, E2-2 > HEB (Langlands et al. 1997). In contrast, Id1 and Id3 bind all the E proteins with similar affinity, with the exception of HEB, which is bound less strongly.

Id proteins also interact with class II bHLH proteins (e.g., OLIG1/2) and non-bHLH TFs (e.g., ternary complex factors (TCFs)), which are a subfamily of ETS-domain TFs (Yates et al. 1999) and paired-domain transcription factor (PAX) family members (Roberts et al. 2001). Thus, in spite of sharing the highly conserved HLH domain, the differences in structure and binding partner preference suggest a broad facet of functional diversity for each Id family member. However, differences in Id-specific binding partners and functionality in CNS cells and especially in endogenous and transplanted NSPCs are poorly understood and need to be further explored for modifying stemcell behavior for a more effective therapeutic repair to ameliorate CNS diseases.

## Regulation of Id abundance

Id gene expression and protein abundance are regulated by a wide range of growth factor and cytokine signaling cascades, as well as by post-translational modifications and degradation. Yet, factors that regulate levels of Id proteins in NSPCs in homeostasis and after CNS injury and disease are just emerging.

### Growth factors

**Fig. 3:**
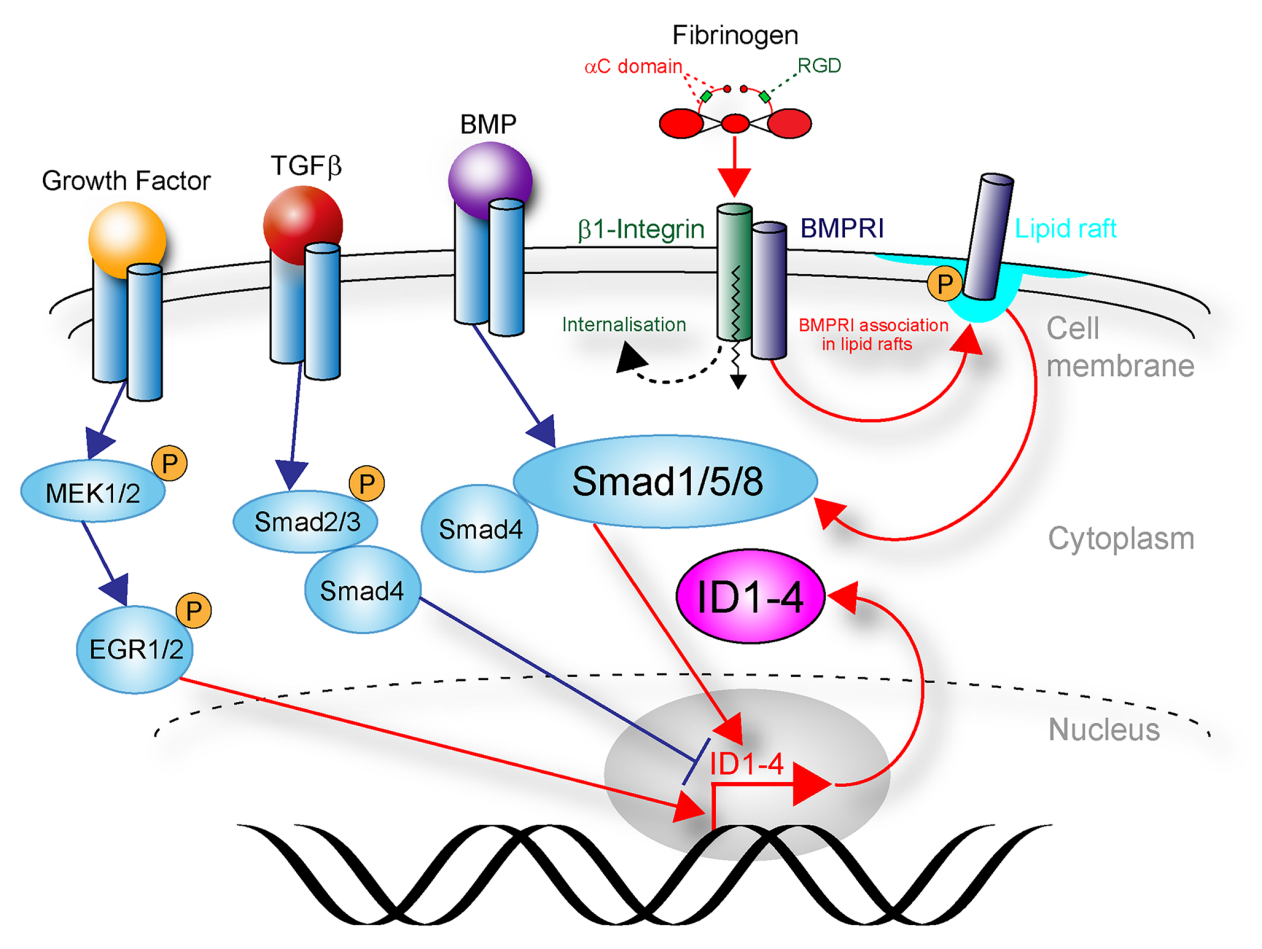
Regulation of Id expression in CNS disease. Bone morphogenetic protein (BMP) induces BMPR I signaling and induces expression of Id1–4 through SMAD binding to BMP-responsive elements in the promoter of Id genes. Fibrinogen (coagulation factor I) induces Id expression via BMPR I signaling activation. Fibrinogen via its integrin interacting αC domain induces BMP type I receptor localization to lipid rafts to activate BMP signaling. Transforming growth factor-β (TGF-β) represses expression of Ids. Receptor tyrosine kinases (such as epidermal growth factor receptor, EGFR and fibroblast growth factor receptor, FGFR), converge on MEK–ERK to activate Id gene transcription via the early growth response (EGR) transcription factor

 In the CNS, *Id* gene expression is regulated by the TGF-β superfamily (Fig. 3) (Hollnagel et al. 1999; Kang et al. 2003; Miyazono and Miyazawa 2002). While TGF-β signaling has opposing effects on Id abundance, BMP signaling robustly increases Id expression in various CNS cell types. We showed that TGF-β represses *Id3* transcription and consequently Id3 protein abundance in adult subventricular zone (SVZ)-derived NSPCs (Bohrer et al. 2015). However, studies in epithelial cells outside the CNS revealed a potential general mechanism of TGF-β-regulated *Id* expression, where the *Id1* expression is transiently induced by TGF-β in a Smad3/4 complex-mediated pathway (Liang et al. 2009), but long-term TGF-β stimulation led to the synthesis of activating transcription factor 3 (ATF3), which interacts with Smad3 and directs the binding of the Smad complex to the CRE/ATF consensus sequence within the *Id1* promoter, mediating the transcriptional repression of *Id1* (Kang et al. 2003). This mechanism has not been reported in CNS cells. The binding of BMP ligands to their corresponding receptors triggers intracellular Smad signaling, mediated by Smad1, a receptor-regulated Smad (R-Smad), and Smad4, a common partner Smad (Co-Smad). The nuclear translocation of the Smad1/Smad4 complex activates the expression of *Id* genes by directly binding to a Smad-binding element (SBE) in their transcription regulatory region (Fig. 3) (Katagiri et al. 2002; Nakahiro et al. 2010). The short-range morphogen BMP is ideally suited to fine-tune stemcell behavior and influencing all stages of neurogenesis. Thus, as part of BMP downstream targets, Id proteins are central in transiting these signals into cellular responses in NSPCs.

In embryonic stem (ES) cells, BMP-triggered Id expression is critical for maintenance of ES cell self-renewal (Hollnagel et al. 1999) (Romero-Lanman et al. 2012; Ying et al. 2003), and BMP-induced Id expression in neural progenitor cells alters the developmental pathway of fetal mouse brain cells from neurogenesis to astrogliogenesis (Nakashima et al. 2001). In the adult neurogenic niche, BMP signaling is finely tuned to support long-term neurogenesis in the subgranular zone and SVZ (Bonaguidi et al. 2008; Mira et al. 2010; Nam and Benezra 2009). Our laboratory showed that, after cortical injury, elevated BMP-2 levels in the adult SVZ stem-cell niche induced rapid and drastic increases in Id3 expression in NSPCs. Consequently, NSPCs with elevated Id3 expression levels preferentially differentiated into SVZ-derived newborn astrocytes (Bohrer et al. 2015).

Recently, we identified the blood-derived coagulation factor fibrinogen as a novel inducer of Id expression in adult SVZ and hippocampal NSPCs. Fibrinogen is enriched in the SVZ stemcell niche environment upon cortical injury due to a leaky stemcell niche vasculature. Fibrinogen deposited in the SVZ stemcell niche environment then induces BMP type I receptor localization to lipid rafts to activate BMP signaling via its integrin interacting αC domain (Pous et al. 2020) (Fig. 3). In addition, fibrinogen deposition in MS lesions triggers Id2 expression in OPCs via the activation of the BMP receptor activin A receptor type I (ACVRI) (Petersen et al. 2017). These findings suggest that vascular pathology with fibrinogen deposition is a general trigger of increased BMP signaling and consequently increased Id abundance that affect glial and neural progenitor cell differentiation (Lin et al. 2021).

The stemcell niche environment is rich in other growth factors, such as fibroblast growth factor-2 (FGF-2) and epidermal growth factor (EGF), that induce *Id* expression through the ERK–MEK pathway in cancer cells by direct binding of early growth responsive protein 1 (Egr-1) to the *Id* promoter (Borlak et al. 2005; Passiatore et al. 2011; Tournay and Benezra 1996). However, the role of FGF-2 and EGF in *Id* gene regulation in CNS cells and, in particular, NSPCs is not yet established.

### Cytokines

 Cytokines regulate Id protein levels in immune cells. In hematopoietic stem cells (HSCs), Id abundance is regulated by various cytokines (e.g., IL-3, IL-6, and GM-CSF) and directs myeloid vs. lymphoid/erythroid cell fate and macrophage vs. neutrophil maturation (Cochrane et al. 2009; Leeanansaksiri et al. 2005; Maeda et al. 2009). Here, the mode of action is activation of C/EBPβ and STAT transcription factors, both of which bind to the enhancer element at the 3′ terminus of *Id* genes (Karaya et al. 2005; Saisanit and Sun 1997; Xu et al. 2003). A role of cytokines in the regulation of *Id* expression in CNS cells and, in particular, NSPCs has not yet been reported.

### MicroRNAs

 Further regulatory modules directly targeting Id expression are microRNAs (miRNAs), such as miR-181a, miR-9, and miR-103 (Amado et al. 2020; Annibali et al. 2012), adding another level to Id regulation. However, regulation of Id expression by microRNAs has been examined mainly in cancer cells. Recently, ablation of the miRNA-17–92 cluster in neural stem cells was found to diminish adult hippocampal neurogenesis and cognitive function, and the miR-17–92 cluster may target the cytoskeleton-associated protein Enigma homolog 1 and its downstream transcription factor Id1 (Pan et al. 2019). However, involvement of microRNAs in the direct control of Id protein levels in NSPCs under physiological and pathological conditions is unknown.

### Id protein regulation

 Id proteins are short-lived. Their half-lives are shorter than 20 min, and they are degraded in the 26S proteasome-dependent pathway after N-terminal ubiquitination. Ubiquitin-dependent degradation of Id1 and Id3 is mediated by the COP9 signalosome (CSN), whereas Id1 and Id3, but not Id2 and Id4, bind to the CSN subunit CSN5 (Berse et al. 2004; Bounpheng et al. 1999; Trausch-Azar et al. 2004). Id interaction with bHLH transcription factors, such as E12, E47, and MyoD, increased Id protein stability, suggesting that Id proteins are less susceptible to degradation by the 26S proteasome when complexed to a bHLH protein (Lingbeck et al. 2005; Trausch-Azar et al. 2004). Phosphorylation of Id2 and Id3 by the cyclin-dependent kinase 2 alters the specificity of the Ids for abrogating both E-box-dependent bHLH homo- or heterodimer complex formation and E-box-dependent reporter gene functions (Deed et al. 1997; Hara et al. 1997) and decreases their stability via the proteasome-dependent degradation (Sullivan et al. 2016). In addition, the C-terminal domains of Id1, Id2, and Id4 contain a destruction box motif (D-box), which is recognized by the anaphase promoting complex/cyclosome (APC/C). Ids interact with the core subunits of APC/C and its co-activator Cdh1 in primary neurons, targeting Ids for degradation (Lasorella et al. 2006). In NSPCs, regulation of Id protein stability remains unclear. A better understanding of Id-specific protein stability and degradation might inform the development of neural stemcell-specific manipulations of Id family members for future therapeutic approaches.

## Id functions in nervous system pathologies

Id protein functions are very well studied in immune cells and in the cancer field (Lasorella et al. 2014; Murre 2019). Id protein expression in the adult CNS is triggered by various extracellular stimuli during pathological conditions. Knowledge of Id function in mature cells in CNS diseases is limited, but a role for the Id protein family in glial and neural precursor cells in CNS disease is emerging. Here, we highlight Id functions in neurological diseases, such as trauma, stroke, MS, Parkinson’s disease (PD), and glioblastoma (GBM).

### Trauma and stroke

 Upregulation of Id genes in a time-dependent manner has been described in astrocytes, oligodendrocyte, and neural progenitor cells after spinal cord injury (Tzeng et al. 2001). However, for a long time, a detailed mechanism of action was lacking. Recently, our laboratory described Id functions in adult NSPCs of the SVZ stemcell niche after traumatic brain injury. In the healthy brain, SVZ NSPCs continuously generate newborn neurons that migrate via the rostral migratory stream to the olfactory bulb to become interneurons. After cortical injury, elevated BMP-2 levels in the adult SVZ stemcell niche are translated into a rapid increase of Id3 expression (Fig. 3). We showed that increased Id3 levels release the E47‐mediated repression of astrocyte‐specific gene expression in adult NSPCs. Consequently, adult NSPCs preferentially differentiate into astrocytes. Id3-mediated astrogenesis from SVZ NSPCs is also triggered by fibrinogen (Fig. 3). Upon cortical photothrombosis, a mouse model for stroke, blood-derived fibrinogen is enriched in the SVZ niche and induces Id3 expression in SVZ NSPCs via activation of the BMP receptor signaling pathway and consequently induces NSPC differentiation into newborn astrocytes (Pous et al. 2020). Overall, these results reveal that the BMP signaling-induced Id3–E47 axis drives SVZ-derived reactive astrocyte contribution to the cortical scar formation after brain trauma. Interestingly, in mature neurons, Id2 expression inhibits E47-induced transcription of the Nogo receptor, which is the key transducer of myelin-mediated inhibition of axonal growth (Lasorella et al. 1996). Furthermore, Akt-mediated phosphorylation of Id2 enhances Id2 protein stability and steers Id2 to the growth cone, where it interacts with radixin that is critical for growth cone formation (Ko et al. 2016). In line with these results, Id2 can be overexpressed in neurons by interfering with its degradation with a D-box mutant Id2 adenoviral vector. This process reduced axon dieback and increased the number and length of regenerative fibers into the lesion gap after spinal cord injury (Yu et al. 2011), suggesting that modifying the Id–E47 balance in NSPCs and neurons might be an attractive route to promote CNS regeneration.

### Multiple sclerosis

 MS is a chronic auto-inflammatory disease of the CNS characterized by blood–brain barrier (BBB) opening, leukocyte infiltration, and demyelination (Lassmann 2018). Insufficient oligodendrocyte precursor cells and the impeded differentiation of mature, myelinating oligodendrocytes are thought to be the major reasons of remyelination failure in MS patients (Franklin and Ffrench-Constant 2017). Similar to developmental stages, oligodendrogenesis in adulthood is regulated by several bHLH TFs, including Olig1 and Olig2 (Lu et al. 2002; Zhou and Anderson 2002), and Id2 and Id4 are key inhibitors of oligodendrocyte differentiation (Kondo and Raff 2000; Marin-Husstege et al. 2006; Wang et al. 2001). Id2 and Id4 directly interact with OLIG1 and OLIG2, inhibiting oligodendrocyte differentiation (Samanta and Kessler 2004). At present, the extracellular signals that control the operation of intracellular inhibitors or timers for oligodendrocyte maturation are not fully understood. In MS, upregulation of Id proteins within an inflammatory demyelinating environment perturbs the remyelination process. Besides increased BMP signaling (Cheng et al. 2007; Samanta and Kessler 2004), OPC contact with myelin increased Id2 and Id4 expression and stopped OPC differentiation (Plemel et al. 2013). The oligodendrocyte-specific G protein-coupled receptor GPR17 is a cell-intrinsic timer of myelination and in an MOG_35–55_-induced experimental autoimmune encephalomyelitis model of MS, the Olig1-regulated GPR17 is upregulated in OPCs in the lesion and blocks oligodendrocyte maturation by increasing Id2 and Id4 expression (Chen et al. 2009). Intriguingly, increased blood-derived fibrinogen deposition in human MS lesions increases Id protein expression in OPCs via the activation of BMP receptor signaling consequently blocking oligodendrocyte differentiation (Fig. 1) (Petersen et al. 2017). Both the Id3-E47-mediated astrocyte differentiation and the Id2/4-Olig1/2-mediated oligodendrocyte differentiation highlight the importance and functional divergence of each Id protein in NSPC lineage commitment. Thus, future therapeutic interventions targeting Id-specific abundance in NSPCs may serve as a potential target for increased myelinating oligodendrocytes in MS lesions and improved myelin repair.

### Parkinson’s disease

 The pathological hallmarks of PD are the progressive degeneration of nigrostriatal midbrain dopaminergic neurons and intraneuronal inclusions of α-synuclein (O’Keeffe and Sullivan 2018; Spillantini et al. 1997). *Id2*-deficient mice display features of PD, such as a decrease of dopaminergic neurons in the olfactory bulb, accompanied by reduced olfactory discrimination (Havrda et al. 2008). While no deficiency of the stem-cell compartment was detected, migrating neuroblasts in *Id2*-deficient mice prematurely undergo astroglial differentiation within a disorganized rostral migratory stream (Havrda et al. 2008). Furthermore, *Id2*-deficient mice showed age-dependent histological alterations in dopaminergic neurons of the substantia nigra pars compacta (SNpC) associated with changes in locomotor activity. Dopamine transporter (DAT) expression was reduced at early ages in *Id2*-deficient mice, and DAT expression was shown to depend on Id2 expression in an in vitro dopaminergic differentiation model. Evidence of neurodegeneration, including activated caspase-3 and glial infiltration, were noted in the SNpC of older *Id2*-deficient mice. These findings document a key role for Id2 in the maintenance of midbrain dopamine neurons (Havrda et al. 2013).

Besides Id2, other Ids might be involved in the regulation of the dopamine system. Id1 and Id3 are induced by dopamine suppression in primary cultured melanotrophs. Continuous stress results in decreased hypothalamic dopaminergic innervation to the intermediate lobe of the pituitary gland, which causes hyperactivation and subsequent degeneration of melanotrophs in the intermediate lobe. These results suggest that the decreased dopamine levels in the intermediate lobe during continuous stress induce Id1 and Id3 expression in melanotrophs. Because Id family members inhibit various bHLH transcription factors, the induced Id1 and Id3 might cooperatively modulate gene expression in melanotrophs under continuous stress conditions to induce hormone secretion (Konishi et al. 2010). These findings suggest that Ids are important in the progression of neurodegenerative disorders involving the dopamine system, such as PD, attention deficit hyperactivity disorder, schizophrenia, and drug abuse.

### Glioblastoma

 GBM is the most aggressive primary brain tumor, and several studies implicated Id family members in disease development. Glioma stem cells, similar to type B adult NSPCs of the SVZ, are defined by their capacity to (1) self-renew in vitro, (2) transplant tumors in vivo, and (3) generate tumors that recapitulate the heterogeneity of the parental tumors (Stiles and Rowitch 2008). The degree to which glioma stem cells resemble neural stem cells and the degree to which the normal lineage hierarchy is maintained in brain tumors are unknown. In the normal neurogenic niche, Id genes maintain self-renewal and multipotency of adult neural stem cells. High Id1 levels mark tumor cells with high self-renewal capacity, whereas low Id1 levels identify tumor cells with proliferative potential but limited self-renewal capacity. Surprisingly, Id1-low cells generate tumors more rapidly and with higher penetrance than Id1-high cells. Moreover, Id1-low cells are characterized by high levels of expression of progenitor-associated markers, including the bHLH transcription factor Olig2. Inhibition of Olig2 but not deletion of Id1 within Id1-low cells significantly prolongs the survival of tumor-bearing mice, underscoring the importance of non-self-renewing lineages in disease progression (Barrett et al. 2012).

Tumor cell invasion is a major contributor to cancer morbidity and is of particular importance in patients with GBM, the highest grade and most aggressive primary brain tumor. In stark contrast to Id1–3, Id4 correlates with survival of glioblastoma patients by decreasing MMP2 expression, a secreted proteinase key for brain tumor invasion, via a direct inhibitory interaction with the bHLH TF Twist1. Structural differences of Id4 might explain its unique function, compared to Id1–Id3 in astrocytic tumors, as Id4 has a unique polyalanine domain at the N-terminus and a polyproline domain at the C-terminus, which could convey specificity for the Id4/Twist1 interaction (Rahme and Israel 2015). Although GBM is an umbrella designation that includes a heterogeneous group of primary brain tumors, GBM exhibits common genetic characteristics. Among these, PDGF/PDGFR-signaling activation appears in nearly 30% of patients and is especially enriched in a pro-neural subtype of GBM (Brennan et al. 2009). Id4 also increases PDGF and nitric oxide synthase 2 (NOS2) expression levels and enhanced the cell self-renewal function in GBM cell lines (Eun et al. 2017). These results place Id4 and its regulatory circuit system into the pole position for regulating the self-renewal and tumor-initiating capacity of GBM stem cells and might provide a promising therapeutic target for GBM.

## Id tools for modifying neural stem-cell behavior

The identification of Id protein function in NSPC fate specification implicates their potential as targets of NSPC manipulation for therapeutic purposes. Here, we describe the major tools to study Id functions in the nervous system: (1) genetic mouse lines with altered Id expression, (2) RNA interference technology, and (3) pharmacological intervention blocking Id-bHLH interaction or affecting Id abundance by blocking or promoting protein degradation (Table 1).

**Table 1:** Genetic mouse lines, tools, and drugs to study Ids in development and disease of the CNS

Tools		Target cell type	Phenotype	References
Germline deletion	Id1^−/−^:Id3^−/−^		Embryonic lethal at E13.5, vascular defects in the forebrain and premature neuronal differentiation	Lyden et al. Nature (1999)
	Id2^−/−^		Defects in adult olfactory neurogenesis and dopaminergic neuron specification	Havrda et al. J Neurosci (2008) Harvda et al. Dis Model Mech (2013)
	Id3^−/−^		Decreased adult SVZ NSPC differentiation into astrocytes after cortical injury	Bohrer et al. EMBO J (2015)
	Id4^−/−^		Perinatal lethality, reduced body size, neural progenitor defected, and defective differentiation of oligodendrocyte lineage	Bedford et al. Dev Biol (2005) Yun et al. Development (2004)
Conditional deletion	Id1^fl/fl^:Id2^fl/fl^:Id3^−/−^:Nestin-Cre-ER^T2^/R26R-YFP	Embryonic and postnatal NSPCs	NSPC detachment from ventricular and vascular niche	Niola et al. Nat Cell Biol (2012)
	Id4^lox/lox^ Rosa26R-CAG:tdTomato mice with GFAP-Cre adenovirus or Id4^fl/fl^:Glast-CreERT2	Adult SGZ NSPCs	Activation of quiescent adult hippocampal stem cells	Zhang et al. Cell Rep (2019) Bloomfield et al. Elife (2019)
Knockdown	Id1 and Id3 siRNA	hESCs hiPSCs	Positive regulation of the hemogenic precursor transition to the hematopoietic lineage	Hong et al. J Cell Sci (2011)
	Id1, Id2, and Id3 siRNA	hiPSCs	Decreased efficiency of hiPSC generation from human dermal fibroblasts	Hayashi et al. PNAS (2016)
Pharmacological inhibition	Peptide-conjugated antisense oligonucleotide (Id-PCAO)	Allograft model of breast cancer	Decreased tumor angiogenesis, tumor growth, and metastasis	Henke et al. Nat Biotechnol (2008)
	Engineered HEB HLH domain (13I)	Neuroblastoma	Impairs complex formation with RB, relieves repression of E protein-activated transcription, inhibition of tumor growth, metastasis, and angiogenesis	Ciarapica et al. Oncogene (2009)
	HLH fragment of MyoD (peptide 3C)	Breast and colon cancer cells	High Id1 affinity. Inhibition of cancer cell proliferation	Chen et al. J Pept Sci (2010)
	Synthetic peptides from Id helix-2 domain (3a and 3b)	Smooth muscle cells	Dysregulated Id1 expression, decreased cell proliferation, and migration	Pellegrino et al. Bioorg Med Chem Lett (2009)
	Peptide aptamer (Id1/3-PA7)	Ovarian and breast cancer cells	Dysregulated Id1 and Id2 expression, induced E-box promoter activity, anti-proliferative and apoptotic effects in cancer cells	Mern et al. Br J Cancer (2010a) Mern et al. Breast Cancer Res Treat (2010b)
	Small molecule inhibitor of USP1 (SJB2-043)	Leukemic cells	Id1–3 degradation, leukemic cell cytotoxicity	Mistry et al. Mol Cancer Ther (2013) Kuang et al. Int J Med Sci (2021)
	Small molecule inhibitors of Id-3 (AGX51)	Endothelial cells/endothelial progenitors	Disrupt Id and E protein interation, leading to the ubiquitinization and degradation of Id1–3, inhibits pathologic ocular neovasculation	Wojnarowicz et al. Cell Rep (2019)

*Id* inhibitor of DNA binding, *NSPC* neural stem/progenitor cell, *PA7* peptide aptamer, *RB* retinoblastoma, *SVZ* subventricular zone

### Genetic mouse lines

 During CNS development, the loss of Id genes leads to premature exit from active cycling and precocious differentiation of progenitor cells (Bedford et al. 2005; Lyden et al. 1999; Yun et al. 2004). Germline deletion of single Id member also reveals their functional divergence in cell-fate determination. In *Id4*-deficient mice, excessive mature oligodendrocytes in the subcortical white matter were found after birth, confirming the function of Id2/4 in oligodendrocyte development (Marin-Husstege et al. 2006). Adult *Id2*-deficient mice revealed no obvious changes in stemcell proliferation in the CNS; however, loss of Id2 results in a Hes1-mediated inhibition of dopaminergic neural differentiation with decreased generation of periglomerular cell layer interneurons in the olfactory bulb and defects in olfactory discrimination (Havrda et al. 2008). Furthermore, *Id2*-deficient mice revealed an age-dependent reduction of dopaminergic neuron numbers of the substantia nigra pars compacta associated with changes in locomotor activity (Havrda et al. 2013).

By characterizing *Id3*-deficient mice, our laboratory established Id3 as a target of blood-derived fibrinogen-induced BMP receptor signaling that determines the differentiation of endogenous NSPC into reactive astrocytes in the SVZ after cortical injury (Bohrer et al. 2015; Pous et al. 2020). With advances in the ability to make genetic modifications, conditional gene deletions allow cell-specific investigations of Id protein functions along temporal and spatial dimensions. Conditional inactivation of NSPC-specific Id1–3 revealed that Id proteins coordinate stemcell activities and triggered detachment of embryonic and postnatal NSPCs from the ventricular and vascular niches, respectively. Interrogation of the gene modules directly targeted by Id deletion in NSPCs revealed that Id proteins repress bHLH-mediated activation of Rap1GAP, the inhibitor of RAP1–GTPase, thus serving to maintain the GTPase activity of RAP1, a key mediator of cell adhesion. By preserving the anchorage of NSPCs to the extracellular environment, Id activity synchronizes NSPC functions to residency in the specialized niche (Niola et al. 2012). Quiescence is essential for the long-term maintenance of adult stem cells, but how stem cells maintain quiescence is poorly understood. Conditional Id4 deletion in hippocampal NSPCs resulted in abnormal accumulation of the bHLH TF Ascl1 and premature stem-cell activation. Id4 sequesters the Ascl1 heterodimerization partner E47, promoting Ascl1 protein degradation and stemcell quiescence (Blomfield et al. 2019).

These recent results highlight the importance of non-transcriptional mechanisms for the maintenance of NSPC quiescence and reveal a role for Id4 as a quiescence-inducing factor, in contrast to its role in promoting the proliferation of embryonic neural progenitors (Blomfield et al. 2019; Zhang et al. 2019). Overall, conditional inactivation of Id genes specifically in NSPCs showed that individual Id family members coordinate different stemcell properties.

### RNA interference technology

 RNA interference (RNAi)-based interventions, such as small interfering RNA (siRNA) and short hairpin RNA (shRNA), have already gained therapeutic applications (Sette et al. 2019). RNA interference technology for knocking down Id expression is broadly used in cancer research (e.g., siRNA targeting Id1 reduces glioblastoma cell invasion and self-renewal (Soroceanu et al. 2013), and implantation of Id2-shRNA knockdown neuroblastoma cells into mice attenuates tumorigenicity, renders the cells immunogenic, and induces host immunity (Chakrabarti et al. 2015)). Id RNA interference technology was successfully applied in adult stemcell niches, where shRNA targeting Id3 regulates terminal differentiation of hippocampal (Micheli et al. 2017) and SVZ NSPCs (Farioli-Vecchioli et al. 2014).

Human pluripotent stemcell (hPSC) research has resulted in clinical applications for various CNS diseases and injuries, and RNA interference technology identified Ids as critical regulator in human embryonic stem cells (hESCs) and induced pluripotent stem cells (hiPSCs). Specific siRNA-mediated downregulation of Id1 and Id3 in either hESC or hiPSC-derived hemogenic precursors resulted in an increase in the generation of mature hematopoietic cells without changing the total cell number and viability of human embryoid bodies (Hong et al. 2011). Moreover, a recent study from Yamanaka’s laboratory proved that siRNA-knockdown of Id1, Id2, or Id3 markedly decreased the efficiency of hiPSC generation from normal human-dermal fibroblasts, but overexpressing any Id gene by retroviral vectors showed an increase in the efficiency of iPSC generation (Hayashi et al. 2016). These results indicate that Id-targeted RNA interference might be a suitable technique to optimize the hPSC technology for therapeutic applications.

### Pharmacological intervention

 Given their various functions in neurological diseases, Id members are attractive therapeutic targets for drug development. Targeted antisense approaches, peptides, and small molecules that can specifically reduce Id family protein abundance and modify Id protein functionality have been proved to be effective in cancer treatment. Indeed, Id protein inactivation by delivery of a peptide-conjugated antisense oligonucleotide, by modulation of Id degradation by the proteasome or by expression of engineered HLH dimerization partner, has been shown to inhibit tumor growth, metastasis and angiogenesis (Chen et al. 2010; Ciarapica et al. 2009; Henke et al. 2008; Kuang et al. 2021; Mistry et al. 2013). Another avenue to modulate the biological function of Id proteins is the development of synthetic molecules that interact with and conformationally perturb the Id HLH dimerization domain. The Id protein surface that recognizes and binds a bHLH protein is built from the parallel packing of helix-1 and helix-2 (Chavali et al. 2001). Consequently, an Id helix-2 peptide mimic was shown to reduce Id1 expression and to modulate smooth muscle-cell proliferation and migration (Pellegrino et al. 2009). Similarly, a novel peptide aptamer, Id1/3-PA7, specifically interacting with Id1 and Id3, was isolated from a randomized combinatorial expression library with yeast and mammalian two-hybrid systems (Mern et al. 2010b). Id1/3-PA7 fused with a cell-protein transduction domain is an anti-tumor agent that triggers cell-cycle arrest and apoptosis in ovarian and breast cancer (Mern et al. 2010a). Moreover, recently, the Benezra laboratory identified the small-molecule pan-Id antagonist AGX51. AGX51 disrupts the Id and E-protein interactions, leading to destabilization of Id proteins and their ubiquitin-mediated degradation. In pre-clinical experiments, AGX51 and derivatives phenocopied the genetic Id loss and inhibited pathologic ocular neovascularization and induced strong anti-tumor effects (Wojnarowicz et al. 2019). Thus, small-molecule compounds, such as AGX51, the first-in-class compound that antagonizes the Id-E-protein interaction formerly considered undruggable, have potential for pharmacological intervention for Id proteins in CNS disease pathogenesis.

## Id proteins as potential targets for modifying neural stemcell behavior

NSPCs hold great promise in physically replacing the damaged tissue after CNS injury and disease (Assinck et al. 2017; Fischer et al. 2020). However, injury-activated endogenous NSPCs predominantly produce scar-forming astrocytes, and the contribution of endogenous or transplanted NSPCs to cell replacement is insufficient for regeneration. We identified rapid and robust upregulation of Id proteins in pathological states with BBB opening as the central player for overriding intrinsic programming of glial and neuronal progenitor cells. Therefore, a targeted therapy against specific Id proteins to modulate the bHLH TF regulatory network might be an attractive and innovative perspective to render NSPCs resistant to the changed environment and to achieve the desired cell fate improving NSPC therapy for cell replacement and better functional restoration.

Indeed, a pioneer study aiming in identifying cellfate determinants after brain injury unraveled the bHLH TF Olig2 as a repressor of neurogenesis. Antagonizing Olig2 function in vivo resulted in a significant number of infected cells that generated immature neurons, not observed after injection of the control virus (Buffo et al. 2005). In addition, overexpression of the bHLH TFs Mash1 and Ngn2 enhanced NSPC survival and promoted robust neuronal differentiation after NSPC transplantation (Yi et al. 2008), and ectopic expression of Olig2 in adult ependymal cells, which are NSPCs in the spinal cord, led to efficient production of oligodendrocytes enabling spinal cord repair (Llorens-Bobadilla et al. 2020). These studies showed that NSPCs engineered via a modification of HLH TFs promote tissue repair after CNS injury or disease. Yet, the functional involvement of Id proteins in endogenous or transplanted NSPCs for cell replacement and CNS repair processes is only scarcely described.

Recently, our laboratory showed that excessive expression of Id3 in SVZ NSPCs after CNS injury scavenges the E47 bHLH transcriptional activity, which suppresses the expression of astrocyte-specific genes including *GFAP* and *GLAST* (also known as *slc1a3*), thus promoting astrogenesis (Bohrer et al. 2015). Excessive expression of Id2 triggered by fibrinogen deposition in demyelinated lesions of MS inhibits the translocation of class II bHLH TFs Olig1/2 into the nucleus, thus blocking the differentiation of OPCs into remyelinating oligodendrocyte (Petersen et al. 2017). Therefore, Id proteins may act as a central hub for translating extracellular signaling into cellular transcriptional properties by counteracting the transcriptional activity of bHLH TFs, and thus are keys in regulating adult NSPC differentiation in CNS injury and diseases.

Moreover, these results revealed that Id2 and Id3 have differential interacting partner preferences towards specific bHLH members, which endow their functional divergence in regulating NSPC fate. Indeed, although highly conserved in the HLH domain, Id2 is structurally different compared to Id3 in the N- and C-terminal parts (e.g., Id2, but not Id3, contains a D-box and nuclear export signal in its C-terminus) (Kurooka and Yokota 2005; Lasorella et al. 2006). Structural and functional differences between Id family members imply the option of modifying specific Id members in a cell- and/or disease-dependent manner.

**Fig. 4:**
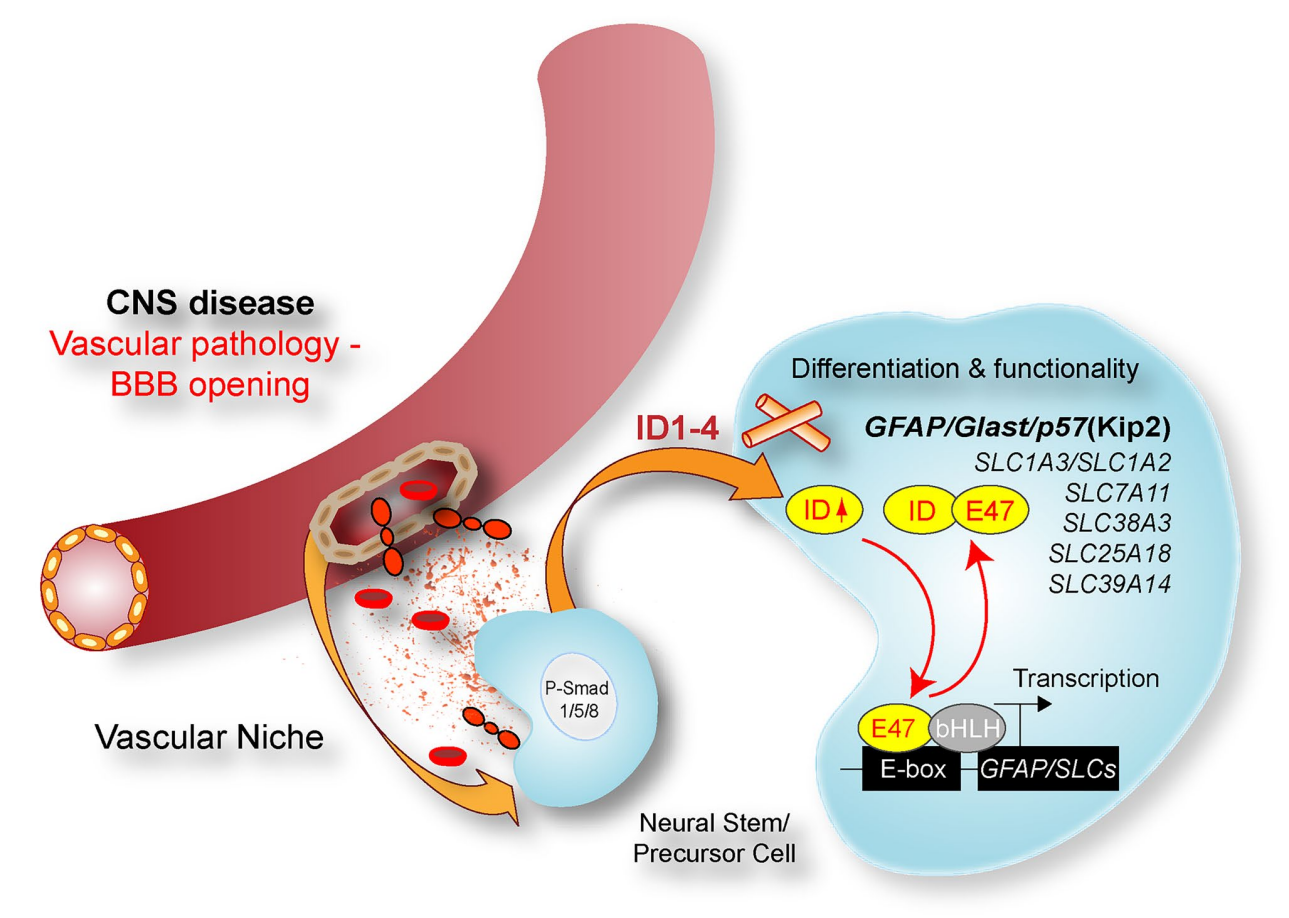
Id function in NSPCs in CNS disease. Cortical injury results in increased SVZ vasculature permeability and fibrinogen deposition into the SVZ stem-cell-niche environment. The deposition of fibrinogen activates BMP signaling and induces phosphorylation of Smad1/5/8 in NSPCs and consequently upregulation of Ids. The Id3‐controlled bHLH transcription factor E47 functioned as a transcriptional repressor of a subset of astrocyte‐specific genes and genes belonging to the solute carrier (SLC) family, including Slc1a3 (GLAST) and Slc1a2 (GLUT1), suggesting a role of Id proteins in regulating cellular homeostasis and metabolism upon environmental alteration

**Fig. 5:**
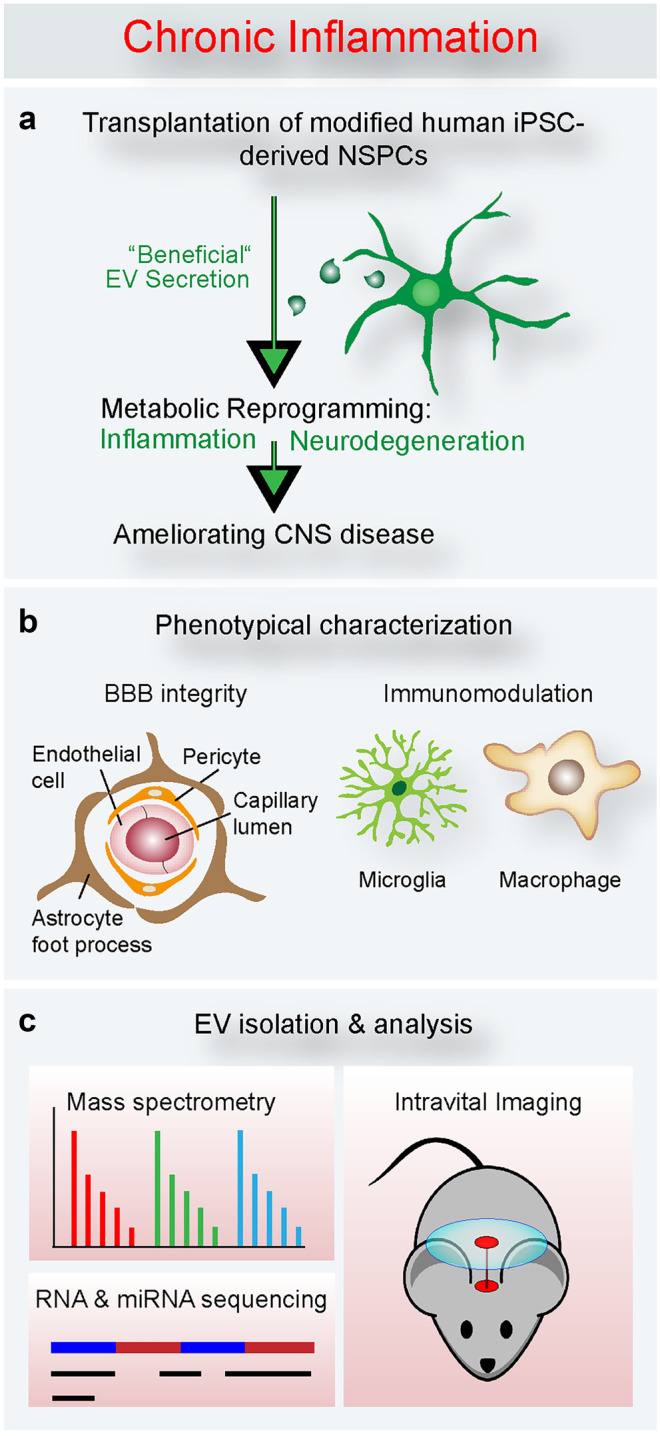
Potential role for Id proteins in modulating NSPC behavior ameliorating CNS diseases. (**a**) Cell transplantation of *Id*-depleted human iPSC-derived NSPCs may result in EV secretion with beneficial effects. (**b**) Phenotypical characterization of *Id*-depleted human iPSC-derived NSPCs on BBB integrity and on myeloid cell activation. (**c**) To discover potential immunomodulatory roles of *Id*-depleted human iPSC-derived NSPCs, EV bio-contents, such as mRNA, miRNA, and protein, can be analyzed by mass spectrometry and RNA sequencing, and EV distribution can be visualized via intravital imaging

NSPCs have immunomodulatory functions, in addition to their function to replace lost tissue. Compelling evidence from experimental animal disease models and early-phase clinical trials identified transplanted NSPCs to act as local “source” for producing and secreting a wide array of neurotrophic and immunomodulatory factors (Martino and Pluchino 2006)(Ziv et al. 2006). Importantly, NSPCs regulate the functional aspects of myeloid cells. NSPCs of the SVZ revealed a secretory protein profile distinct from other brain cells modulating microglial activation, proliferation, and phagocytosis. NSPC-derived VEGF was necessary and sufficient to exert at least some of these effects in mice (Mosher et al. 2012). NSPCs secrete extracellular vesicles, which encase proteins and microRNAs that act as morphogens to preferentially target microglia regulating their morphology and immune responses (Morton et al. 2018). Additionally, under chronic inflammatory condition, NSPCs ameliorate neuroinflammation by detecting the extracellular succinate released by mononuclear phagocytes. The uptake of extracellular succinate, which is released by the inflammatory mononuclear phagocyte, upregulates the succinate co-transporter of the SLC family in the NSPCs and triggers the secretion of prostaglandin E2 by the NSPCs with consequential anti-inflammatory effects (Peruzzotti-Jametti et al. 2018). Interestingly, our study identified that the Id–E47 axis regulates, besides *GLAST*, several other members of the SLC family, such as *Slc1a2*, *Slc25a18*, *Slc38a3*, *Slc39a14*, and *Slc7a11* (Bohrer et al. 2015) (Fig. 4). SLC family members are broadly involved in glutamate transport, intracellular ionic balance, and vesicle transport, suggesting a potential function of Id signaling in directing cellular homeostasis and metabolism in NSPCs upon environmental alteration (Lin et al. 2021). In addition, Id abundance regulates cytokine release, such as VEGF, GROα (also known as CXCL1), and IL-8 (Fontemaggi et al. 2009; Jin et al. 2011; Lasorella et al. 2005; Lin et al. 2021). Therefore, modifying Id expression in endogenous or transplanted NSPCs may lead to an altered cellular functionality and cytokine/EV release, potentially reducing neuroinflammation and neurodegeneration (Fig. 5). To this end, using CRISPR/Cas9 technology and small-molecule inhibitors to delete Id proteins may help address this issue without affecting important functions of the BMP signaling pathway in stem-cell proliferation and differentiation.

## Concluding remarks

Ids are potent regulators of glial and neural stemcell behavior in different CNS injuries and diseases, such as stroke, trauma, MS, PD, and GBM. Ids are unstable, rapidly regulated proteins that hinder the bHLH transcription factor DNA-binding activity by modifying its regulatory network. Although Id proteins were formerly considered undruggable, recent developments in small-molecule compounds regulating Id-E-protein interactions allow control of Id levels in glial and neural stem cells. Thus, pharmacological intervention involving Id proteins has potential to modify stemcell behavior and ameliorate CNS disease. Several key issues remain to be resolved. Optimal Id targeting requires a greater understanding of the cell types and injury states in which Id family members and their interaction partners are co-expressed and their mode of interactions. Over the next few years, we expect that research will elucidate the function of the individual Id proteins in CNS disease and their underlying molecular mechanisms, the identification of unique interaction sites for Id targeting, and how individual Id depletion alters the stemcell functionality.
